# Plant Traits Demonstrate That Temperate and Tropical Giant Eucalypt Forests Are Ecologically Convergent with Rainforest Not Savanna

**DOI:** 10.1371/journal.pone.0084378

**Published:** 2013-12-17

**Authors:** David Y. P. Tng, Greg J. Jordan, David M. J. S. Bowman

**Affiliations:** School of Plant Science, University of Tasmania, Hobart, Australia; University of New South Wales, Australia

## Abstract

Ecological theory differentiates rainforest and open vegetation in many regions as functionally divergent alternative stable states with transitional (ecotonal) vegetation between the two forming transient unstable states. This transitional vegetation is of considerable significance, not only as a test case for theories of vegetation dynamics, but also because this type of vegetation is of major economic importance, and is home to a suite of species of conservation significance, including the world’s tallest flowering plants. We therefore created predictions of patterns in plant functional traits that would test the alternative stable states model of these systems. We measured functional traits of 128 trees and shrubs across tropical and temperate rainforest – open vegetation transitions in Australia, with giant eucalypt forests situated between these vegetation types. We analysed a set of functional traits: leaf carbon isotopes, leaf area, leaf mass per area, leaf slenderness, wood density, maximum height and bark thickness, using univariate and multivariate methods. For most traits, giant eucalypt forest was similar to rainforest, while rainforest, particularly tropical rainforest, was significantly different from the open vegetation. In multivariate analyses, tropical and temperate rainforest diverged functionally, and both segregated from open vegetation. Furthermore, the giant eucalypt forests overlapped in function with their respective rainforests. The two types of giant eucalypt forests also exhibited greater overall functional similarity to each other than to any of the open vegetation types. We conclude that tropical and temperate giant eucalypt forests are ecologically and functionally convergent. The lack of clear functional differentiation from rainforest suggests that giant eucalypt forests are unstable states within the basin of attraction of rainforest. Our results have important implications for giant eucalypt forest management.

## Introduction

The study of ecotones between forest and open vegetation has been central to the development of ecological and evolutionary theory [1–5]. Such vegetation transition zones may provide insights into global change biology [6,7]. In particular, they provide model systems to investigate how extrinsic factors (e.g. fire, soils and climate) [8–10] and intrinsic processes such as biological feedbacks [11–13] contribute to the dynamics of ecosystems. They are therefore particularly important for testing contemporary ecological theories such as Alternative Stable States models [14,15]. 

**Figure 1:**
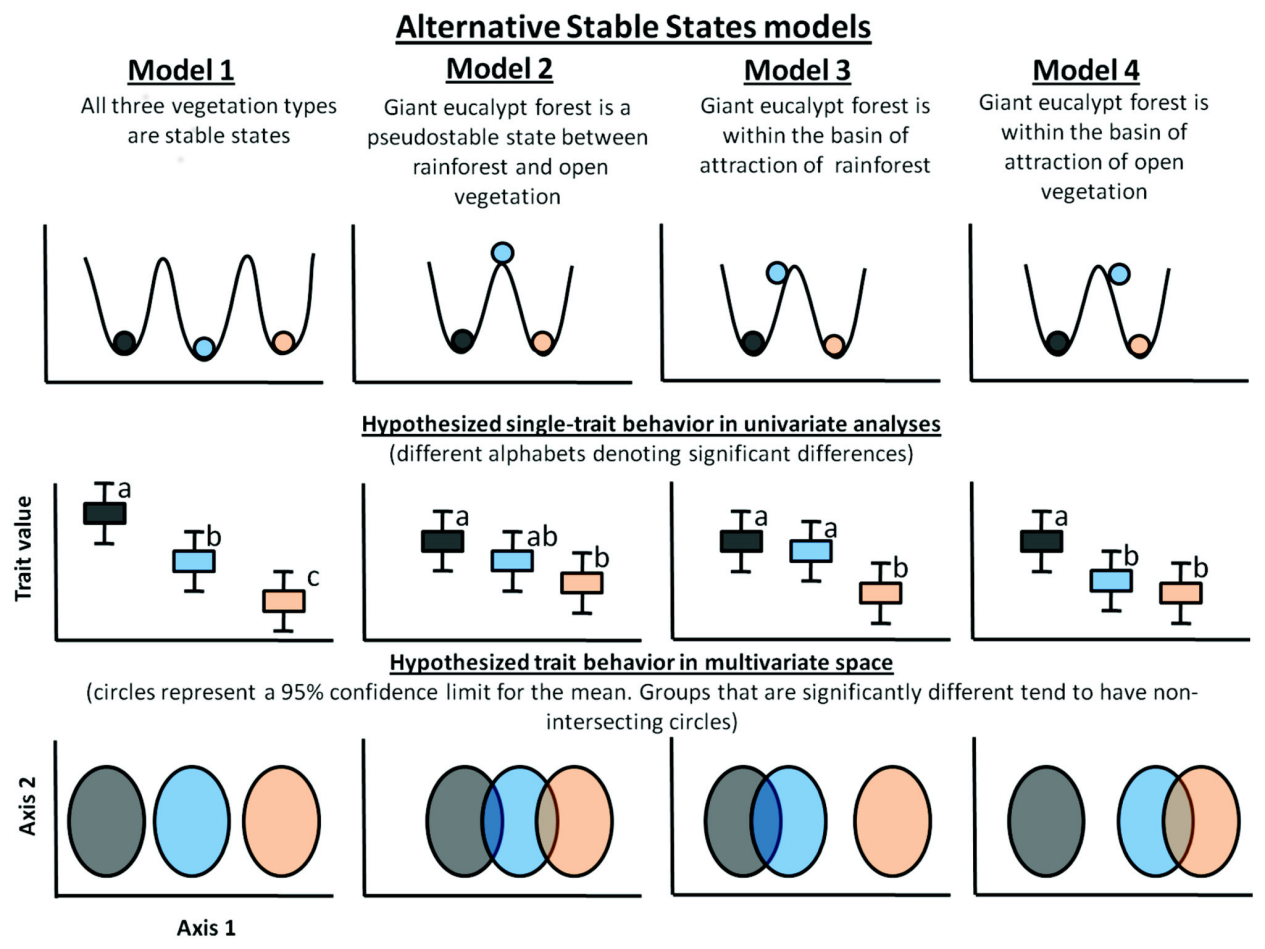
Idealised Alternative Stable States ‘ball and cup’ scenarios for rainforest (dark grey), giant eucalypt forest (blue) and open vegetation (orange) and their corresponding hypothesized trait behavior in univariate and multivariate analysis outputs. In each case, the overlap between to the confidence limits of each functional profile will denote the functional affinities between habitats.

Alternative Stable States models are becoming increasingly useful in explaining ecological dynamics, with empirical evidence for their existence at scales ranging from species assemblages [16,17] to biomes [18]. These models suggest that many ecosystems exist as stable states and are often depicted as “balls” that lie in “basins” or domains of attraction in a three-dimensional ‘stability landscape’. The depth of the “basins” denotes the stability of the ecosystems [19–21] (Figure 1). These models differ from classical succession models in which ecosystems slide along a continuum of steady states [14]. 

Alternative Stable States systems therefore arise from interactions between extrinsic and intrinsic factors. Changes in extrinsic factors, such as climate and fire [22–24], tend to drive changes in ecosystems, including transitions from one stable state to another. However, the stable states only exist when intrinsic characteristics of the ecosystem generate positive feedbacks that create and maintain stability [25–29]. For example, in fire-susceptible regions different ecosystems may occur as alternative stable states because of different fire regimes caused by differences in fuel load, flammability, microclimate or other factors. In such instances the characteristics of the organisms in each ecosystem contribute to creating different fire regimes. Measuring targeted functional traits [30] of the component organisms of ecosystems is therefore an obvious way to test whether these ecosystems represent alternative stable states, as these traits can be of great significance in plant function, community assembly and ecological processes [29–31]. For example, leaf mass per unit area (LMA), a commonly studied functional trait, is correlated with potential relative growth rate or mass-based maximum photosynthetic rate, leaf lifespan, leaf defences, etc. [30], and has been shown to be significantly different across the forest – savanna divide [32,33]. 

**Figure 2:**
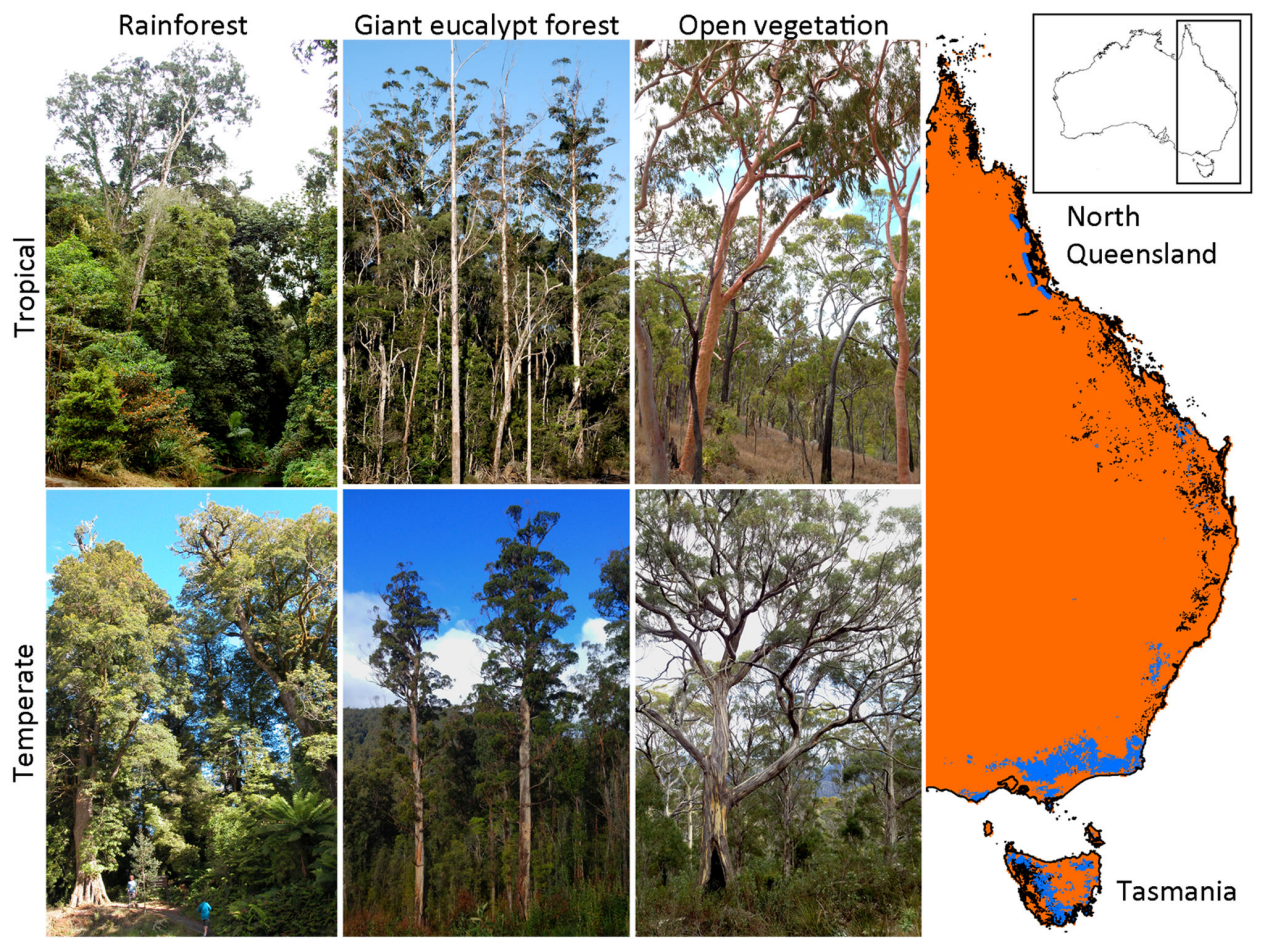
The distribution of rainforest (black) and giant eucalypt forest (blue) along the east coast of the Australian continent. The orange-coloured regions are open vegetation (including savanna and open eucalypt woodland). The ecotonal nature of giant eucalypt forest is most pronounced in tropical north Queensland, where giant eucalypt forests form narrow bands between rainforest and savanna (spatial extent exaggerated for clarity), and in cool temperate Tasmania, where giant eucalypt forests form a broad transition between the west and the eastern parts of the island. The inset images feature representative rainforests, giant eucalypt forests and open vegetation of the tropical and temperate zones. Note the taller stature and open canopy of giant eucalypts relative to rainforest in the understoreys.

The east coast of mainland Australia and Tasmania presents an excellent geographical setting to macroecologically study forest-open vegetation transitions within a single continent. From the tropics to the temperate zone, rainforests exist as disjunct patches within a matrix of eucalypt-dominated savanna or open woodland [34,35] (Figure 2). Giant eucalypt forests (also locally known as tall open forests, wet sclerophyll forests or mixed forests) dominated by eucalypt species that can attain heights exceeding 70m, are often observed wedged in the ecotone between rainforest and savanna or open canopy vegetation [36]. In the tropics, these giant eucalypt forests are dominated by *Eucalyptus grandis* W. Hill ex Maiden and range from a few hundred meters to a few kilometres wide in extent [37,38] while in temperate zones in Victoria and Tasmania, similar forests dominated by a range of species (e.g. *E. regnans* F. Muell., *E. obliqua* L'Hér.) may predominate over several kilometres [36,39]. Although these forests have no species in common, there are phylogenetic links between these geographical regions, evidenced by the presence of shared genera and subgenera. These forests include the world’s tallest angiosperms [36], are home to several important threatened species [38], and represent major carbon sinks [40,41]. Together, giant eucalypt forests and rainforests have been the focus of major conflicts between ecological and economic interests because they are major forestry resources and extensive areas have been cleared for agriculture [42]. In temperate Australia, logging of these forests is ongoing.

Warman and Moles [14] hypothesized that the tropical *E. grandis* forests are unstable states forming an ecotone between rainforest and savanna (Figure 1). By contrast, Wood & Bowman [43] inferred that temperate giant eucalypt forests in Tasmania are stable states, but of lower stability (i.e. occupying a shallower basin of attraction; Figure 1) than the adjacent temperate rainforest and open vegetation. However, it remains unclear whether these tropical and temperate systems are functionally convergent, and whether it is possible to create a unified Alternative Stable States model for these geographically distant, but ecologically similar systems [36]. Several authors have argued that the eucalypt dominants of these forests are essentially rainforest successional species [36,44,45]. However, these forests have largely been viewed as discrete vegetation types distinct from rainforest due to the subjective vegetation classifications based on the eucalypt dominants (i.e. Model 1; Figure 1). A sound landscape ecology theory augmented by functional trait based understanding of the ecology of these giant eucalypt forests is necessary for effective management of these dynamic ecosystems. If these forests are functionally convergent with each other across tropical and temperate regions, and if they are indeed unstable ecological states (sensu Warman and Moles [14]), the traditional approaches to their ecological management and conservation will need revision.

Adopting a macroecological approach, we test whether the functional traits of trees and shrubs found in the rainforest/open vegetation transitions in both tropical and temperate regions are consistent with the patterns expected if Alternative Stable State theory applies to these vegetation (Figure 1). We also test whether the giant eucalypt forests of the tropical zone and the temperate zone are functionally convergent. First, we define state scenarios under an Alternative Stable States context, for rainforests, giant eucalypt forests and open vegetation (Figure 1). Within both temperate and tropical regions, we expect that giant eucalypt forest will fall under one of four possible models: (Model 1) it forms a third discrete stable state; (Model 2) it is an unstable state intermediate between the stable states of rainforest and open vegetation; (Model 3) it is unstable and falls within the basin of attraction of rainforest, or; (Model 4) it is unstable and falls within the basin of attraction of open vegetation types (Figure 1). Second, we use univariate analyses to compare each functional trait across vegetation types and multivariate analyses to visualize and compare the functional profile for each vegetation type (Figure 1). In addition, the proximity of giant eucalypt forest species from both regions in multivariate space will indicate the degree of functional convergence. This is the first study to explicitly link functional trait behavior and Alternative Stable States models in Australian terrestrial ecosystems (see also Dantas et al. [33]). 

## Materials and Methods

### Ethics Statement

Permission to sample vegetation was obtained from the Queensland Government Environmental Protection Agency (permit number WITK07872410) for North Queensland sites, and the Department of Primary Industries, Parks, Water and Environment (permit number FL12268) for Tasmanian sites. The field studies did not involve threatened or endangered species.

### Study Sites And Sample Collection

We sampled rainforest, and the surrounding giant eucalypt forest and open vegetation but did not sample treeless grasslands or sedgelands in two regions: tropical north Queensland and cool temperate Tasmania. North Queensland experiences a humid tropical climate with a typical site (Herberton: 17°38′S, 145°39′E) having a mean maximum annual temperature of 27.1°C and a mean annual rainfall of 2240 mm. The climate is thermally aseasonal, but has a summer-rainfall bias [46]. The regions of Tasmania studied here experience a cool temperate climate with a mean maximum annual temperature of 18.4°C and a mean annual rainfall of 2070mm for a typical site (Arve Valley: 43°14′S, 146°79′E). The climate is thermally seasonal and has winter-dominated precipitation [46]. In each region the three vegetation types are readily recognised, allowing for *a priori* allocation of vegetation samples and species; (i) rainforests have closed canopies and an absence of eucalypts; (ii) giant eucalypt forests are emergent above either rainforest, or a mix of shrubby and grassy understoreys, and; (iii) open vegetation is dominated by shorter eucalypts and has shrubs and herbaceous (including grass) species tolerant of high light environments. Tropical open forests/woodlands have a well developed grassy understorey and are classified as tropical savannas. Open vegetation in the temperate region is referred to here as savanna, as they can have some structural similarities with tropical eucalypt savannas. In both regions, the tree and shrub species measured for functional traits (Table 1) were selected on the basis of their relative abundance in at least one of the localities, with the aim of capturing a representative spread of species in all three vegetation types. While many of the species sampled were widespread within their thermal zone, the trait data for any given species were taken from specimens collected from only one locality. The few species that occurred in more than one vegetation type were only sampled in the vegetation type where they occurred at the highest abundance. This selection process, based on extensive fieldwork to indentify species and assess their community affinities, was designed to minimise the confounding effect of giant eucalypt forest at different successional stages having varying components of rainforest species. Although vines were common in the tropical vegetation types, they were not sampled for trait measurements because of their low representativeness in temperate rainforest and giant eucalypt forest, and also because not all the functional traits used for our tree and shrub species will be applicable to vines.

**Table 1:** Functional traits selected for the current study and their functional significance relevant to the current study. A reference set for each trait is compiled.

**Functional Trait**	**Unit**	**Functional significance of relevance to current study**	**Refs**
***Leaf Traits***			
Delta 13 C (δ^13^C)	‰	Correlated to plant water use efficiency and may also segregate plants of different successional status.	1
Leaf Area	mm^2^	Consequential for leaf energy and water balance. Interspecific variation in leaf size has been connected with climatic variation, where heat stress, cold stress, drought stress and high radiation all tend to select for relatively small leaves.	2
Leaf mass per area (LMA)	g m^-2^	Correlated with potential relative growth rate. Higher values correspond with high investments in structural leaf defences and leaf lifespan, but also slower growth.	3
Leaf Slenderness	Unitless	Involved in control of water and temperature status. Slender leaves have a reduced boundary layer resistance and are can thus regulating their temperature through convective cooling more effectively.	4
***Bole Traits***			
Wood density	g cm^-3^	Positively correlated with drought tolerance and tolerance of mechanical or fire damage; related to stem water storage capacity, efficiency of xylem water transport, regulation of leaf water status and avoidance of turgor loss.	5
Maximum height	M	Positively correlated with competitive ability of plants.	6
Bark thickness	Unitless	Correlated to fire resistance with thicker bark expected in fire prone areas.	7

1 [73,80,81]

2 [61, 63, 74]

3 [69, 82; 83]

4 [84, 85]

5 [76, 86–90]

6 [51, 69, 91]

7 [57,92,93]

At the following three localities near the western edge of the Wet Tropics World Heritage Area we sampled the three vegetation types: Davies Creek (17°08′S, 145°22′E), Mt Baldy (17°17′S, 145°25′E) and Paluma (18°56′S 146°10E). At each site the rainforest was the simple notophyll vine forest type [47]. *Eucalyptus grandis* dominated the giant eucalypt forest, and at all three localities, the understorey exhibited the full range of variability of being grassy-shrubby to being dominated by mesophytic broadleaved trees. The savanna was dominated in different localities by different eucalypt species (*Eucalyptus crebra* F. Muell., *E. Mediocris* L.A.S. Johnson & K.D. Hill, *E. tereticornis* Sm., *E. tindaliae* Blakey) with grassy or shrubby understoreys [48]. We sampled 32, 22 and 16 species from rainforest, giant eucalypt forest, and savanna respectively (Appendix S1 and Table S1 in Appendix S1). For the most part, species were exclusive to one vegetation type.

Field sampling in Tasmania was undertaken in cool temperate rainforest and giant eucalypt forest from the northeast (41°14′S 147°44′E), southeast (42°56′S 147°17′E) and southern localities (43°05′S 146°43′E). This widespread sampling allowed us to sample the full structural range of cool temperate rainforest types (sensu Jarman et al. [49]) associated with the two dominant giant eucalypt species, *Eucalyptus regnans* and *E. obliqua* [50]. These rainforests are dominated by some combination of *Nothofagus cunninghamii* (Hook.) Oerst., *Atherosperma moschatum* Labill. and *Anodopetalum biglandulosum* (Hook.) Hook.f. The more patchy distribution and lower species richness of cool temperate rainforest and the broad extent of giant eucalypt forest necessitated a slightly different protocol than used in tropical Queensland. For the giant eucalypt forests, we restricted our sampling to areas dominated by *Eucalyptus regnans* or *E. obliqua*. Open woodland (savanna) adjacent to rainforest and giant eucalypt forest was geographically restricted at the northeastern and southern sampling sites. Because the suite of savanna species and their dominant overstorey eucalypts are common and geographically widespread in Tasmania, it was decided that sampling species of this vegetation type from southeastern localities was sufficient to obtain a representative Tasmanian sample. This savanna vegetation was dominated by *Eucalyptus pulchella* Desf. with *E. viminalis* Labill. co-dominants and a shrubby understorey. We sampled 15, 23 and 20 species from temperate rainforest, giant eucalypt forest and savanna, respectively (Appendix S1 and Table S1 in Appendix S1). 

For each species sampled, we measured and compiled functional trait data on at least four to five mature (> 60% potential height) individuals per species. We measured a set of four leaf traits and three bole traits (Table 1), following methods outlined by Cornelissen et al. [30]. These traits are related to shade-tolerance, light use efficiency, water use efficiency, drought tolerance, nutrient use, growth rate, and fire resistance [30] (See also Table 1 and references therein). For leaf carbon isotope ratio (δ^13^C) determination, the leaves of four to five individuals were bulked, ground finely and δ^13^C assessed by the School of Plant Biology, University of Western Australia. For leaf area and leaf mass per area (LMA), two to 20 replicates per individual of sun-exposed leaves were obtained from the tree or shrub mid-canopy. For species with compound leaves, leaflets were taken to be the functional unit equivalent to leaves. For shrubs and short trees, an extension cutter was used to obtain the leaves but for trees taller than 10 meters, canopy branches were collected using a slingshot and weighted line. Only fully expanded leaves were used and these were scanned with a flatbed scanner and the leaf scans were processed by imaging software ImageJ to obtain leaf areas. Leaf slenderness was measured as the ratio of the leaf length to leaf breadth. These leaves were then dried to a constant weight at 60°C and weighed. LMA was then determined by dividing leaf dry weight by the leaf area. For wood density, we followed a protocol similar to Falster & Westoby [51]. For trees, we collected branches and obtained two to five 5cm segments of the branch approximately 1m from the branch tip, whereas for shrubs, we collected wood segments by destructive sampling from the base of the shrub. The bark was removed from the wood segments and the displacement method was used to obtain the branch segment fresh volume. The branch segments were then dried at 60°C for a week, weighed, and the wood density calculated as dry weight divided by fresh volume. Maximum height (Ht_max_) was obtained from literature sources [52–56]. Bark thickness was only measured on trees, and was obtained using a bark gauge at a height of 1.3m above the ground. In trees with fissured bark, we took readings from ‘ridges’ inbetween fissures, and in individuals with buttresses, we took readings from the trunk above the buttresses. We excluded this trait for shrubs because it was not possible to obtain bark thickness values for this life form in the same standardized way that we could for trees. As bark thickness increases with bole diameter, we expressed bark thickness relative to stem diameter (e.g. Lawes et al. [57]) by multiplying bark thickness by two and dividing this figure by the recorded diameter. We therefore sampled bark thickness from 26, 16 and 9 tree species from tropical north Queensland, and 8, 16 and 6 tree species from temperate Tasmania from their respective rainforests, giant eucalypt forests and savannas. 

### Data Analysis

All variables were checked for normality and where required were log-transformed. For each region, univariate one-way ANOVAs were performed for each trait. Significant differences between habitats were determined by Tukey HSD tests using a confidence level of 0.05. All univariate analyses were performed in R. We also undertook univariate phylogenetic ANOVAs on each functional trait (Appendix S1). The results were essentially similar to the normal set of ANOVAs (Table S2 in Appendix S1) and so we report only the latter. Two-way factorial ANOVAs using regions (tropical and temperate), vegetation type (rainforest, giant eucalypt forest and savanna) and their interaction were also performed. We excluded bark thickness for the two-way ANOVA as data for this trait was only available for trees. 

For the multivariate analyses, we used canonical variate analysis to visualize overall trait position within and among habitats. This method is a weighted ordination method in which axes are weighted to maximise the difference between *a priori* groups of multivariate observations [58,59]. MANOVA is the multivariate analogue of ANOVA, and tests for differences among groups. We performed both one-way and two way MANOVAs and *post-hoc* pair-wise tests using a confidence level of 0.05 were used to test for differences between groups. These multivariate analyses were performed using the discriminant analysis function in JMP 10.0.0 (SAS Institute, Inc., Cary, NC). As with the two-way factorial ANOVAs, bark thickness was excluded from the multivariate analysis as we only had measurements for tree species.

## Results

### Univariate Analyses

**Figure 3:**
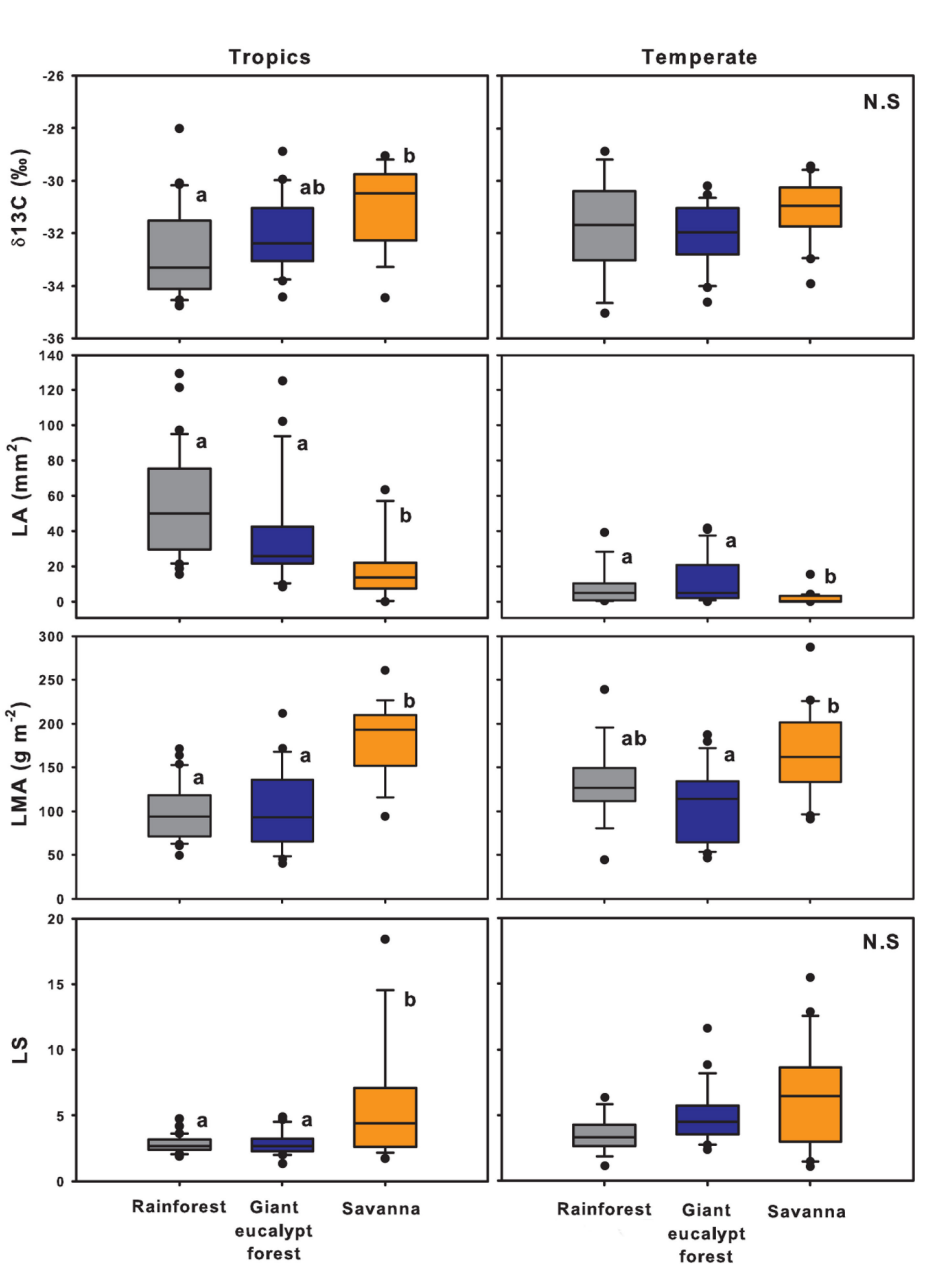
Boxplots showing the leaf trait behavior of rainforest (grey), giant eucalypt forest (blue) and savanna (orange) species from the tropical north Queensland (left block) and the cool temperate Tasmania (right block). Shown are carbon isotope composition (δ^13^C), leaf area (LA), leaf mass per area (LMA) and leaf slenderness (LS). Each box encompasses the 25th to 75th percentiles; the median is indicated by the boldest vertical line and the other vertical lines outside the box indicate the 10th and 90th percentiles. Dots indicate outliers. One-way ANOVAs were performed on the data (log-transformed for LA and LS) and significant differences between vegetation types are indicated by different letters based on Tukey HSD tests at a 0.05 confidence level (see Methods; Table 2). N.S denotes non-significance.

**Figure 4:**
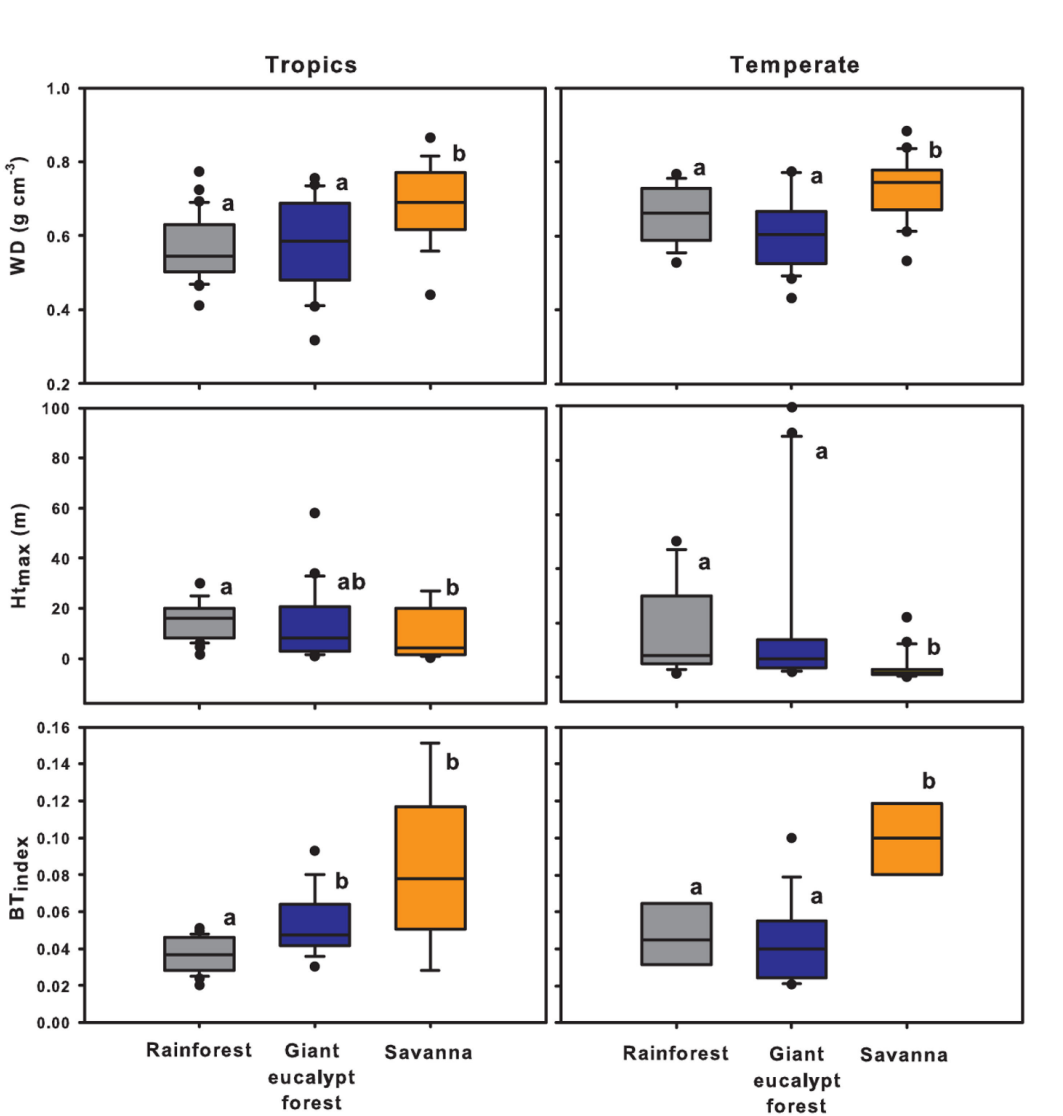
Boxplots showing the bole trait behavior of rainforest (grey), giant eucalypt forest (blue) and savanna (orange) species from the tropical north Queensland (left block) and the cool temperate Tasmania (right block). Shown are wood density (WD), maximum height (Ht_max_), and bark thickness index (BT_index_). Each box encompasses the 25th to 75th percentiles; the median is indicated by the boldest vertical line and the other vertical lines outside the box indicate the 10th and 90th percentiles. Dots indicate outliers. One-way ANOVAs were performed on the log-transformed data (except WD) and significant differences between vegetation types are indicated by different letters based on Tukey HSD tests at a 0.05 confidence level (see Methods; Table 2).

The two-way ANOVAs all showed significant differences, often with significant interaction effects, so we performed one-way ANOVAs. These showed a number of differences, and a number of similarities in trait behavior in both regions (Table 2; Figure 3, 4). In the tropical system, rainforest and savanna were significantly different in all traits, with the latter having a significantly higher δ^13^C ratio, LMA, leaf slenderness, wood density and bark thickness, but lower leaf area and maximum height than the former (Figure 3, 4). For most traits giant eucalypt forest was not significantly different from rainforest, with the exception of greater bark thickness. 

**Table 2:** One-way ANOVA results for of carbon isotopes ratios (δ^13^C), leaf area, leaf mass per area (LMA), leaf slenderness, wood density, maximum height, and bark thickness index comparisons between rainforests, giant eucalypt forests and savannas of tropical and temperate regions.

**Functional Trait**	**Tropical Queensland**		**Temperate Tasmania**		**Both Regions**	
	***F*_2,67_**	***P***	***F*_2,55_**	***P***	***F*_5,122_**	***P***
*Leaf traits*						
δ^13^C	6.97	0.0018**	2.45	0.09 (N.S)	4.73	0.0005***
Leaf area	16.31	<0.0001***	10.13	0.0002***	34.04	<0.0001***
LMA	20.56	<0.0001***	9.04	0.0004***	14.98	<0.0001***
Leaf slenderness	11.48	<0.0001***	2.58	0.08 (N.S)	7.54	<0.0001***
*Bole traits*						
Wood density	7.77	0.0009***	10.29	0.0002***	9.71	<0.0001***
Maximum height	4.88	0.011*	15.11	<0.0001***	9.67	<0.0001***
^*^Bark thickness	17.31	<0.0001***	9.15	0.0009***	NA	NA

Leaf area, leaf slenderness, Maximum height, and bark thickness were log transformed before Analysis. S denotes non-significance. Bark thickness was left out in the analysis with both regions combined as data for this trait was only available for tree species. *Bark thickness measurements were only performed on trees, hence the different degrees of freedom (Tropical Queensland: *F*_2,48_; Temperate Tasmania: *F*_2,27_) from the other traits.

In the temperate system, δ^13^C ratios and leaf slenderness were not significantly different across vegetation types, but leaf area and maximum height were significantly greater, while wood density and bark thickness were significantly lower for rainforest than savanna species (Figure 3, 4). However, temperate rainforest and savanna were not significantly different in LMA. Temperate giant eucalypt forest was not significantly different from rainforest in any of the measured traits.

### Multivariate Analyses

**Figure 5:**
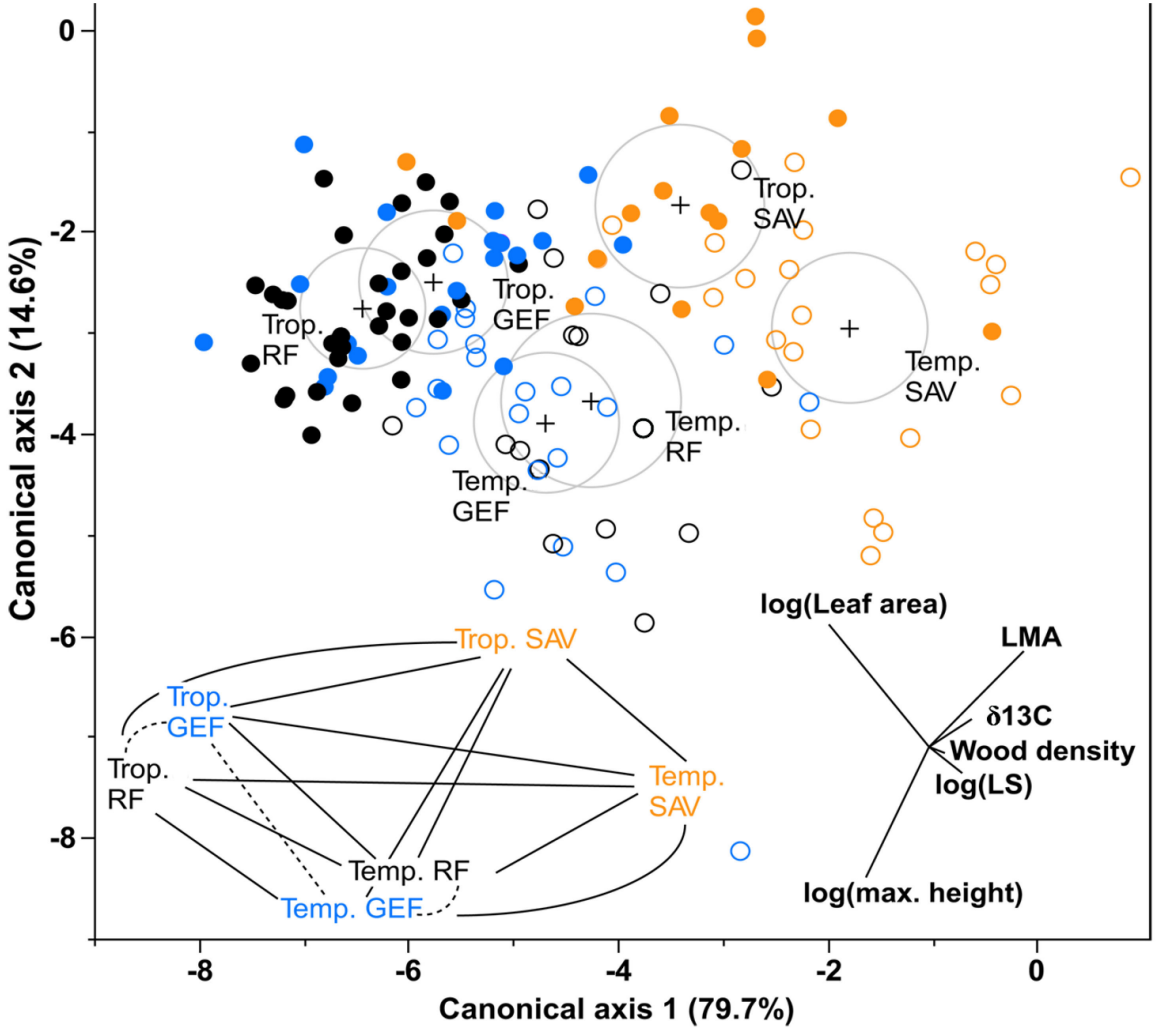
Canonical variate analyses of functional trait means of 128 species from tropical and (closed circles) temperate (open circles) rainforest (black), giant eucalypt forest (blue) and savanna (orange). Six functional traits were used: carbon isotopes (δ13C); leaf area; leaf mass per area (LMA); leaf slenderness (LS); wood density, and; maximum height plotted into multivariate space. Each dot represents a species. On the bottom right the trait weightings (transformed where required) are plotted onto the graphs as vectors whose length and direction represent the contribution of the variable in explaining the clustering pattern. For each vegetation group, each multivariate mean is visualized as large grey circles encircling a black cross, the size of which corresponds to the 95% confidence limit for the mean. Groups that are significantly different tend to have non-intersecting circles. The proximities and overlaps of these circles are used to corroborate trait behavior with Alternative Stable States model scenarios (Figure 1). The bottom left inset is the results of pairwise *post-hoc* tests of a one-way MANOVA where unbroken lines represent significant differences between vegetation types, and dashed lines represent non-significance.

Two-way MANOVAs show that region (Wilks' Lambda: *F*_6,117_ = 19.53, *P* < 0.0001), vegetation type (Wilks' Lambda: *F*_12,234_ = 13.45, *P* < 0.0001), and region × vegetation type interactions (Wilks' Lambda: *F*_12,234_ = 1.87, *P* < 0.038) were significant. We therefore performed one-way MANOVAs which showed highly significant differences among vegetation types within the tropics (*F*_2,67_ = 27.33, *P* < 0.0001) and the temperate zone (*F*_2,55_ = 6.54, *P* = 0.003), and in the combined analysis (*F*_5,122_ = 14.5, *P* < 0.0001). *Post-hoc* pairwise-tests show that the major differences occurred between rainforest and savanna in both regions, and also across regions (Figure 5). Tropical rainforest was also significantly different from temperate rainforest, and tropical savanna from temperate savanna (Figure 5). However, tropical and temperate giant eucalypt forests were not significantly different (Figure 5). 

Likewise in canonical variate analyses for the individual regions, significant differences were found between vegetation types within both the tropical (Wilks' Lambda: *F*_12,124_ = 6.85, *P* < 0.0001) and temperate regions (Wilks' Lambda: *F*_12,100_ = 7.92, *P* < 0.0001). When all six habitats are analysed together, the canonical variate analyses was also significant (Wilks' Lambda: *F*_30,470_ = 8.88, *P* < 0.0001). As the trends of the individual regional analyses are captured in the combined analysis, we present only the plot for the combined ordination (Figure 5). In multivariate space, the spread of species show clear clustering of rainforest and giant eucalypt forest species and this is visualized by the overlapping 95% confidence limit circles (Figure 5). δ^13^C, LMA, leaf slenderness and wood density appear to be the major variables segregating the tropical and temperate savannas from the rainforest and giant eucalypt forest as a whole (Figure 5). However, by virtue of their positioning in multivariate space, the temperate rainforest cluster, whilst being most functionally akin to giant eucalypt forest, also exhibits a mild clustering with the savanna cluster. The overlap of the rainforest and giant eucalypt forest clusters was stronger within each region, and the tropical giant eucalypt forest appear to be converging with the temperate giant eucalypt forest and temperate rainforest clusters. In contrast, the tropical and temperate rainforest regions are diverging, largely on the basis of tropical rainforest species having greater leaf area and temperate rainforest exhibiting higher leaf slenderness. LMA, and to a lesser extent δ^13^C and wood density, are responsible for the segregation of the two savannas from the rainforest-giant eucalypt forest cluster, but both savannas are also clearly segregated.

## Discussion

Our univariate and multivariate analyses of leaf and bole functional traits of representative plants from rainforest and savanna in temperate and tropical Australia show differences consistent with what we would expect to find in the context of rainforest and savanna being alternative stable states [4,5]. Our results also provide direct support that the giant eucalypt forests are functionally closer to rainforests than to savanna, and therefore better thought of as a successional stage towards rainforests. The basis and significance of these hypotheses are outlined below.

### Tropical And Temperate Rainforest And Savanna

Tropical and temperate rainforests were functionally divergent (Figure 5), and this is augmented by the similar results obtained from both phylogenetic (Table S2 in Appendix S1) and normal ANOVAs (Table 2). Leaf area was generally larger in tropical systems than in temperate systems as expected [60–64]. This is consistent with well-known differences in physiognomy [60] and phylogenetic origins [65] of the rainforest types. Experimental work by Lusk et al. [66] and Xiang et al. [67] show trade-offs for traits like LMA, leaf area and other leaf traits between tropical and temperate rainforest, and this might explain the tropical-temperate rainforest functional divergence. Collectively this suggests that rainforest is not a cohesive functional entity across the Australian continent, apart from the unifying factor of having a closed canopy [68]. 

There were marked leaf and bole trait differences between rainforest and savanna vegetation. Our results supported the concept that savanna plants will have relatively thicker bark than rainforest trees [57]. LMA, which correlates strongly with important leaf physiological and structural functions such as growth rate, leaf lifespan, etc. [30,69,70] (Table 1), was higher in both temperate and tropical savanna than their rainforest counterparts, reflecting intrinsic biological differences between savanna and rainforest. Consistent with this interpretation is the finding of Hoffman et al. [32] that LMA is a key functional trait explaining the differences between forest-savanna congeneric species pairs in central Brazilian ecosystems.

In the tropics three traits related to water relations (δ^13^C, leaf slenderness and wood density) showed strong difference between rainforest and savanna, but δ^13^C and leaf slenderness were not significantly differentiated across temperate rainforest boundaries. Consistent both with the literature [71,72] and the concept that water use efficiency is related to water availability, was our finding that tropical savanna species have more positive δ^13^C, and therefore higher water use efficiency [73] than rainforest species. Tropical savanna species had slender leaves probably because narrow leaf width is related to radiative cooling in dry climates [74,75]. Higher savanna wood density relative to rainforest is probably due to the higher potential of savanna species for tolerating drought stress [76]. 

### Giant Eucalypt Forests

The multivariate ANOVAs and canonical variates analyses show that overall: (i) temperate and tropical giant eucalypt forests are functionally convergent, and; (ii) temperate and tropical giant eucalypt forests are closer in function to their respective rainforests than to their respective savannas (Figure 5). Even though there was high variability in species traits and overlaps in functional profile, the segregation between savannas and rainforests/giant eucalypt forests was significant (Figure 5). Augmenting these interpretations, we also obtained similar results for both phylogenetic (Appendix S1) and normal ANOVAs (Table 2) for most of the traits tested.

For all traits except bark thickness, univariate analyses showed that giant eucalypt forest were not significantly different from their respective rainforests. Significantly, in both temperate and tropical giant eucalypt forests, LMA did not differ from their respective rainforests but was markedly different from their respective savannas, suggesting that the trees and shrubs of giant eucalypt forest on a whole are more functionally akin to rainforest in their leaf functioning. However, LMA in temperate rainforest was not significantly different from savanna unlike in the tropics (Figure 3). This could be an inherent effect of thermal differences between the two regions, which may also explain why δ^13^C and leaf slenderness were not significantly different across temperate rainforest boundaries, unlike in the tropics (Figure 3) [67].

Bark thickness was the only trait in the tropics that deviated from our hypothesized model that giant eucalypt forest is functionally different from savanna but not from rainforest (Figure 1). This indicates that the trees in the tropical giant eucalypt forest show some affinity to tropical savanna in their degree of fire-tolerance, and contrasts with the temperate system which supports model scenario 3. The narrower spatial extent of the ecotone in tropical Queensland relative to the temperate one [38,39] (Figure 2) could be a plausible explanation, as plants in the narrower tropical ecotone might be more prone to frequent low-intensity fires and therefore exhibit a greater degree of fire-adaptation. We acknowledge that more data, which was beyond our capacity to collect, on postfire recovery traits (e.g. resprouting, serotiny) would help further illuminate the relationship between savannas, giant eucalypts forests and rainforests.

The co-occurrence of rainforest and giant temperate eucalypt forest species to create distinctive vegetation types (‘mixed forests’) has long been recognised [44], but the status of tropical communities dominated by giant eucalypts has been controversial [14]. Our findings demonstrate that giant eucalypt forests in both the temperate and tropical regions are functionally more similar to rainforest than to savanna, which can lend support to the idea that these eucalypt forests lie within the basin of attraction of rainforest (Model 3 in Figure 1). The convergence of the functional trait profiles of tropical and temperate giant eucalypt is consistent with insights from restoration ecology, which show that within a successional sequence, trait composition exhibits a clear decrease in multivariate distance with increasing restoration age, indicating trait convergence through time, regardless of whether species convergence occurs [77]. For these reasons giant eucalypt forest species can be considered early to mid successional rainforest species (i.e. secondary forest species) corroborating both Schimper’s [78] early view that giant eucalypt forests are essentially rainforests, and our proposition that giant eucalypts are long-lived emergent rainforest pioneer trees [36]. The view that giant eucalypt forest is successional to rainforest would also explain the well documented tendency for their understoreys to accumulate rainforest species [9,38,50], thereby resulting in a two-tiered rainforest where the successional species (i.e. the giant eucalypts) form the overstorey [34,36]. The reason for the development of rainforest developing beneath eucalypts relates to differences in shade tolerance of species growing in these communities: eucalypts and rainforest pioneers are well known for being shade intolerant [34,36], while primary rainforest species are usually shade tolerant [34]. This major physiological difference results in the dominance of eucalypts in the high light environments of recently burnt stands, and the inability of eucalypts to regenerate in unburnt stands. Rainforest species are able to continually establish under dense regenerating giant eucalypt stands [36].

With the obvious exception that giant eucalypt forests have greater statures than rainforests in both regions, the functional trait profile of the sampled giant eucalypt forest species was essentially the same as that of the sampled rainforest species (Figure 4, 5). This suggests that while giant eucalypts (*E. grandis* and *E. regnans*) are often the focal point for classifying these forests [38,50], their heights contribute little to the overall functional profile of the forest. The contribution of height to the ability of these individual species to compete successfully against other plants and dominate these transitional zones is consistent with the view that these plants are true ecotonal specialists [36].

While our study examined giant eucalypt forests in tropical and temperate regions, forests of the giant eucalypt *E. diversicolor* F. Muell. exist in the Mediterranean-climate zone of western Australia. These western Australian giant eucalypt forests differ from those on the Australian east coast in the total absence of rainforest species, due to the extinction of rainforests from that region over the last 10 million years [36,79]. Functional trait studies could be used to investigate if these forests can be interpreted as a stable state alternative (and hence rainforest analogue) to other open woodland types (e.g. dominated by *Eucalyptus marginata* Donn ex Sm.) in this region. Forests dominated by other very large (exceeding 50m height) eucalypt species also occur in subtropical zones associated with rainforests in Southeast Queensland and New South Wales [34] and there is also scope for testing ideas related to functional traits in an alternative stable state context in these systems.

While our study has adopted a broad conceptual approach by constructing the functional profile of the sampled vegetation types from species that occur typically in those vegetation types across an entire region, there are inherent differences between the tropical and temperate systems that go beyond those that can be captured in our functional trait study (such as succession patterns, gap dynamics, and the role of functional groups that are not present in both areas). These differences could be more effectively captured by including more functional traits [30] or by using an ecophysiological approach. At a more local scale, there is also scope for modelling the shifts in functional profiles with successional age and understanding the functional thresholds in the transition from rainforest to savanna. Such approaches could involve modelling trait profile discontinuities against a canopy closure index (i.e. Dantas et al. [33] to examine specific rainforest-savanna transitions under different environmental settings. In such studies we would recommend more consideration of traits relating to regeneration and growth strategies.

In conclusion, our study bridges landscape ecology theory and plant functional biology by examining the functional traits of representative tree and shrub species from tropical and temperate rainforest – giant eucalypt forest – savanna transitions. Functional leaf and bole trait segregation between rainforest and savanna were clear, especially in the tropics. The giant eucalypt forests however were functionally more akin to rainforest than to savanna in both tropical and temperate regions. These results augment the suggestion that giant eucalypts such as *E. grandis* and *E. regnans* are essentially rainforest trees [36] and calls for a functional, rather than floristic classification of these giant eucalypt forests. We expect this work to have important implications for the management and conservation of these unique giant eucalypt forests, and also encourage more landscape ecology – plant functional trait syntheses in terrestrial ecosystems.

## Supporting Information

Appendix S1 **Method of phylogenetic correction for univariate traits, data analysis and trait data.**
**Table S1.** Species mean trait values of carbon isotope ratios (δ^13^C, ‰), leaf area (LA, mm^-2^), leaf mass per unit area (LMA, g m^-2^), leaf slenderness (LS), wood density (WD, g cm^-3^), maximum height (Ht_max_, meters) and bark thickness (BT_index_) for 128 species collected from rainforest (RF), giant eucalypt forest (GEF) and savanna (SAV) in Queensland and Tasmania. For maximum height, some of the species values compiled from literature but some were reduced in accordance with our field observations. For bark thickness, we only have data for 81 tree species.
**Table S2.** Phylogenetic One-way ANOVA results for leaf and bole plant functional trait comparisons between rainforests, giant eucalypt forests and savannas of tropical and temperate regions. Bark thickness was excluded from this analysis as it consisted of only a subset of the species in the phylogenetic tree.
(DOCX)
