# Supplementary material for: Plant Traits Demonstrate That Temperate and Tropical Giant Eucalypt Forests Are Ecologically Convergent with Rainforest Not Savanna
Source: PLoS One. 2013 Dec 17;8(12):e84378. doi: 10.1371/journal.pone.0084378 (PMC3866143; doi:10.1371/journal.pone.0084378)
Supplement: Appendix S1 — Method of phylogenetic correction for univariate traits, data analysis and trait data. Table S1. Species mean trait values of carbon isotope ratios (δ13C, ‰), leaf area (LA, mm-2), leaf mass per unit area (LMA, g m-2), leaf slenderness (LS), wood density (WD, g cm-3), maximum height (Htmax, meters) and bark thickness (BTindex) for 128 species collected from rainforest (RF), giant eucalypt forest (GEF) and savanna (SAV) in Queensland and Tasmania. For maximum height, some of the species values compiled from literature but some were reduced in accordance with our field observations. For bark thickness, we only have data for 81 tree species. Table S2. Phylogenetic One-way ANOVA results for leaf and bole plant functional trait comparisons between rainforests, giant eucalypt forests and savannas of tropical and temperate regions. Bark thickness was excluded from this analysis as it consisted of only a subset of the species in the phylogenetic tree. (DOCX) [file pone.0084378.s001.docx]

**APPENDIX S1.** Method of phylogenetic correction for univariate traits, data analysis and trait data.

As species descend hierarchically from common ancestors, statistical independence of the trait data cannot be assumed and we endeavoured to account for phylogenetic effects in our analyses. A phylogenetic tree for the study species (Table S1) was constructed using Mesquite ver. 2.75 based on the maximally resolved supertree for angiosperms [1]. As this tree was not fully resolved, genus- and species-level polytomies were resolved by obtaining additional phylogenetic information from (i) the user-supplied data repository in Phylomatic for the Ericaceae [2], Myrtaceae [3], and Proteaceae [4] and (ii) from searches of the primary literature for the following clades: the genera *Acacia* [5], *Archirhodomyrtus* and *Rhodomyrtus* [6], *Eucalyptus* [7]. As the phylogenetic tree was a composite from multiple sources and we lacked data on branch lengths, all branch lengths were set equal to 1.

A set of univariate one-way ANOVAs (Table S2) subject to phylogenetic corrections was performed for the dataset using the phylanova function in the phytools R package. To determine which groups were significantly different, *post hoc* tests were carried out as part of the phylanova function, which performs a Bonferroni correction on the data. These results (Table S2) were similar to the normal ANOVAs which are presented in the manuscript.

**Table S1.** Species mean trait values of carbon isotope ratios (δ^13^C, ‰), leaf area (LA, mm^-2^), leaf mass per unit area (LMA, g m^-2^), leaf slenderness (LS), wood density (WD, g cm^-3^), maximum height (Ht_max_, meters) and bark thickness (BT_index_) for 128 species collected from rainforest (RF), giant eucalypt forest (GEF) and savanna (SAV) in Queensland and Tasmania. For maximum height, some of the species values compiled from literature but some were reduced in accordance with our field observations. For bark thickness, we only have data for 81 tree species.

| **Taxon** | **Veg.** | **δ^13^C** | **LA** | **LMA** | **LS** | **WD** | **Ht_max_** | **BT_index_** |
| --- | --- | --- | --- | --- | --- | --- | --- | --- |
| Tropical Queensland |  |  |  |  |  |  |  |  |
| *Acronychia acronychioides* | RF | -33.20 | 44.24 | 116.03 | 3.16 | 0.58 | 15 | 0.050 |
| *Alangium villosum* | RF | -33.39 | 27.20 | 67.84 | 2.70 | 0.54 | 20 | 0.027 |
| *Alphitonia whitea* | RF | -31.52 | 74.91 | 163.81 | 2.84 | 0.58 | 20 | 0.040 |
| *Archirhodomyrtus beckleri* | RF | -30.14 | 15.58 | 118.50 | 2.52 | 0.53 | 7 | 0.073 |
| *Brackenridgea australiana* | RF | -33.77 | 49.88 | 114.84 | 3.53 | 0.77 | 6.5 | 0.031 |
| *Cardwellia sublimis* | RF | -30.08 | 76.19 | 110.69 | 3.47 | 0.56 | 20 | 0.025 |
| *Casearia costulata* | RF | -32.65 | 21.22 | 90.41 | 3.05 | 0.65 | 6 | 0.035 |
| *Castanospora alphandii* | RF | -34.18 | 52.13 | 97.93 | 3.59 | 0.63 | 17 | 0.024 |
| *Croton triacros* | RF | -32.37 | 44.88 | 99.17 | 2.43 | 0.57 | 10 | 0.043 |
| *Daphnandra repandula* | RF | -34.24 | 40.33 | 48.98 | 3.64 | 0.50 | 9 | 0.047 |
| *Darlingia darlingiana* | RF | -31.26 | 97.35 | 149.57 | 4.20 | 0.69 | 23 | 0.027 |
| *Doryphora aromatica* | RF | -34.55 | 45.49 | 76.03 | 2.95 | 0.55 | 18 | 0.040 |
| *Eupomatia laurina* | RF | -34.54 | 75.65 | 69.45 | 2.58 | 0.50 | 7 |  |
| *Ficus leptoclada* | RF | -33.32 | 18.95 | 76.94 | 2.50 | 0.48 | 13 | 0.030 |
| *Flindersia brayleyana* | RF | -31.36 | 84.97 | 171.32 | 2.00 | 0.52 | 25 | 0.030 |
| *Flindersia pimenteliana* | RF | -30.37 | 22.95 | 93.81 | 2.40 | 0.50 | 25 | 0.020 |
| *Geissois biagiana* | RF | -33.38 | 129.51 | 103.30 | 2.07 | 0.46 | 25 | 0.025 |
| *Guioa lasioneura* | RF | -33.49 | 23.59 | 128.83 | 2.75 | 0.72 | 9.5 | 0.041 |
| *Hedycarya loxocarpa* | RF | -34.76 | 74.66 | 68.75 | 2.41 | 0.51 | 1.6 |  |
| *Litsea connorsii* | RF | -32.34 | 29.60 | 118.35 | 2.66 | 0.50 | 16 | 0.031 |
| *Macaranga subdentata* | RF | -31.05 | 50.26 | 82.92 | 3.50 | 0.58 | 14 | 0.038 |
| *Myrsine porosa* | RF | -34.75 | 36.31 | 80.65 | 2.72 | 0.68 | 8 | 0.046 |
| *Neolitsea dealbata* | RF | -33.48 | 86.03 | 102.20 | 2.32 | 0.47 | 20 | 0.048 |
| *Pittosporum wingii* | RF | -34.13 | 36.36 | 60.34 | 3.02 | 0.67 | 10 | 0.044 |
| *Polyosma alangiacea* | RF | -32.05 | 59.94 | 90.62 | 2.67 | 0.63 | 20 | 0.051 |
| *Polyscias australiana* | RF | -33.55 | 63.09 | 64.38 | 2.54 | 0.41 | 16 | 0.046 |
| *Sloanea langii* | RF | -31.81 | 76.66 | 153.16 | 1.89 | 0.53 | 20 | 0.042 |
| *Steganthera laxiflora* | RF | -34.49 | 64.02 | 71.09 | 2.46 | 0.50 | 7 | 0.047 |
| *Symplocos cochinchinensis* | RF | -32.06 | 121.45 | 127.06 | 2.38 | 0.57 | 22 | 0.030 |
| *Tasmannia insipida* | RF | -34.08 | 24.67 | 74.69 | 4.75 | 0.57 | 4.5 |  |
| *Toona ciliata* | RF | -28.02 | 66.40 | 61.96 | 2.12 | 0.53 | 30 | 0.029 |
| *Alstonia mulleriana* | GEF | -32.66 | 58.60 | 95.54 | 2.97 | 0.67 | 20 | 0.041 |
| *Breynia stipitata* | GEF | -33.82 | 13.57 | 85.73 | 1.99 | 0.73 | 3.5 |  |
| *Callicarpa pedunculata* | GEF | -32.60 | 48.13 | 45.32 | 2.81 | 0.69 | 1.3 |  |
| *Cryptocarya vulgaris* | GEF | -32.14 | 27.04 | 125.89 | 2.37 | 0.70 | 12 | 0.046 |
| *Duboisia myoporoides* | GEF | -34.42 | 22.74 | 56.12 | 4.11 | 0.32 | 6 | 0.093 |
| *Endiandra discolor* | GEF | -31.19 | 23.37 | 131.48 | 2.30 | 0.58 | 8 | 0.064 |
| *Eucalyptus grandis* | GEF | -30.06 | 26.28 | 156.09 | 4.70 | 0.59 | 58 | 0.030 |
| *Eucalyptus macta* | GEF | -29.94 | 24.05 | 211.40 | 4.88 | 0.63 | 34 | 0.041 |
| *Euroschinus falcatus* | GEF | -31.04 | 40.42 | 76.95 | 2.45 | 0.45 | 25 | 0.044 |
| *Glochidion sumatranum* | GEF | -32.64 | 36.93 | 90.08 | 2.71 | 0.58 | 16 | 0.062 |
| *Guioa acutifolia* | GEF | -32.16 | 31.13 | 137.28 | 3.03 | 0.74 | 8 | 0.064 |
| *Litsea leefeana* | GEF | -33.17 | 74.28 | 95.30 | 2.02 | 0.49 | 14 | 0.049 |
| *Lophostemon suaveolens* | GEF | -30.74 | 32.27 | 171.59 | 2.34 | 0.57 | 30 | 0.038 |
| *Macaranga involucrata* | GEF | -31.51 | 102.12 | 66.89 | 1.31 | 0.42 | 3 |  |
| *Melicope elleryana* | GEF | -32.94 | 125.07 | 61.41 | 2.31 | 0.41 | 23 | 0.075 |
| *Pomaderris argyrophylla* | GEF | -31.00 | 23.88 | 158.68 | 3.06 | 0.76 | 5 | 0.043 |
| *Psychotria loniceroides* | GEF | -33.28 | 9.52 | 88.87 | 3.27 | 0.69 | 1 |  |
| *Rhodomyrtus canescens* | GEF | -33.64 | 12.34 | 99.12 | 3.23 | 0.69 | 1.7 |  |
| *Schizomeria ovata* | GEF | -31.32 | 21.26 | 135.62 | 2.49 | 0.54 | 8 | 0.044 |
| *Trema tomentosa* | GEF | -33.02 | 21.79 | 59.04 | 2.60 | 0.46 | 2.5 | 0.071 |
| *Wikstroemia indica* | GEF | -32.69 | 8.36 | 39.75 | 3.59 | 0.58 | 2.5 |  |
| *Wilkiea pubescens* | GEF | -28.88 | 25.62 | 90.67 | 2.09 | 0.60 | 4 |  |
| *Acacia calyculata* | SAV | -30.47 | 5.77 | 209.56 | 7.22 | 0.77 | 1.56 |  |
| *Acacia flavescens* | SAV | -32.82 | 63.54 | 137.88 | 3.40 | 0.80 | 9 | 0.146 |
| *Banksia aquilonia* | SAV | -32.62 | 13.82 | 213.62 | 13.60 | 0.72 | 20 | 0.078 |
| *Corymbia leptoloma* | SAV | -29.06 | 24.24 | 192.81 | 4.59 | 0.63 | 19 | 0.064 |
| *Eucalyptus crebra* | SAV | -31.89 | 14.62 | 260.48 | 6.75 | 0.70 | 27 | 0.085 |
| *Eucalyptus mediocris* | SAV | -30.52 | 16.32 | 217.65 | 3.41 | 0.69 | 15 | 0.069 |
| *Eucalyptus tereticornis* | SAV | -30.62 | 29.38 | 195.14 | 6.94 | 0.62 | 27 | 0.037 |
| *Eucalyptus tindaliae* | SAV | -31.94 | 19.00 | 207.51 | 4.41 | 0.61 | 24 | 0.028 |
| *Ficus opposita* | SAV | -33.00 | 55.65 | 120.86 | 2.25 | 0.44 | 4 |  |
| *Hakea plurinervia* | SAV | -29.35 | 19.81 | 195.81 | 5.80 | 0.72 | 1.6 |  |
| *Hibbertia melhanioides* | SAV | -30.27 | 3.99 | 164.54 | 3.06 | 0.59 | 1.2 |  |
| *Hibbertia stirlingii* | SAV | -29.65 | 0.17 | 139.46 | 11.60 | 0.61 | 0.35 |  |
| *Persoonia falcata* | SAV | -34.47 | 13.50 | 209.19 | 18.39 | 0.63 | 3.7 | 0.152 |
| *Petalostigma pubescens* | SAV | -29.22 | 9.43 | 189.21 | 1.74 | 0.77 | 1.02 |  |
| *Pomaderris canescens* | SAV | -30.25 | 13.88 | 163.81 | 2.61 | 0.78 | 2.05 |  |
| *Pultenaea millarii* | SAV | -29.84 | 0.57 | 93.67 | 2.56 | 0.87 | 1.14 |  |
| *Syncarpia glomulifera* | SAV | -30.22 | 10.57 | 163.69 | 2.40 | 0.63 | 20 | 0.087 |
|  |  |  |  |  |  |  |  |  |
| Temperate Tasmania |  |  |  |  |  |  |  |  |
| *Anodopetalum biglandulosum* | RF | -31.69 | 4.58 | 105.95 | 2.76 | 0.64 | 15 | 0.043 |
| *Anopterus glandulosus* | RF | -29.46 | 39.20 | 142.73 | 4.26 | 0.59 | 10 | 0.069 |
| *Aristotelia peduncularis* | RF | -34.39 | 10.32 | 44.23 | 2.60 | 0.77 | 4 |  |
| *Atherosperma moschatum* | RF | -28.89 | 8.36 | 116.10 | 2.63 | 0.57 | 45 | 0.026 |
| *Cenarrhenes nitida* | RF | -32.75 | 20.98 | 165.71 | 3.94 | 0.73 | 10 | 0.051 |
| *Eucryphia lucida* | RF | -31.05 | 5.01 | 126.25 | 3.70 | 0.59 | 30 | 0.031 |
| *Leptospermum laenigerum* | RF | -31.14 | 0.50 | 119.81 | 3.12 | 0.63 | 30 | 0.047 |
| *Nothofagus cunninghamii* | RF | -30.39 | 1.16 | 130.00 | 1.14 | 0.53 | 50 | 0.032 |
| *Olearia persoonioides* | RF | -32.03 | 3.53 | 153.75 | 2.67 | 0.73 | 4 |  |
| *Orites diversifolia* | RF | -33.71 | 10.46 | 149.40 | 5.55 | 0.75 | 8 |  |
| *Tasmannia lanceolata* | RF | -29.40 | 8.06 | 112.05 | 4.02 | 0.57 | 8 |  |
| *Telopea truncata* | RF | -31.84 | 11.21 | 238.80 | 6.36 | 0.67 | 8 |  |
| *Trochocarpa cunninghamii* | RF | -33.03 | 0.34 | 142.86 | 2.39 | 0.66 | 1.5 |  |
| *Trochocarpa disticha* | RF | -35.04 | 0.92 | 104.56 | 4.63 | 0.67 | 5 |  |
| *Trochocarpa gunnii* | RF | -31.64 | 0.47 | 111.38 | 3.32 | 0.67 | 7 |  |
| *Acacia dealbata* | GEF | -31.01 | 0.04 | 89.85 | 5.74 | 0.48 | 33 | 0.023 |
| *Acacia melanoxylon* | GEF | -30.19 | 13.12 | 134.42 | 4.78 | 0.54 | 30 | 0.045 |
| *Acacia verniciflua* | GEF | -32.16 | 4.67 | 93.74 | 7.22 | 0.62 | 8 | 0.041 |
| *Bedfordia salicina* | GEF | -32.79 | 29.55 | 87.94 | 5.77 | 0.65 | 7 | 0.041 |
| *Cassinia trinervia* | GEF | -33.91 | 2.76 | 46.21 | 11.59 | 0.52 | 7 |  |
| *Coprosma quadrifida* | GEF | -32.58 | 1.01 | 51.57 | 3.58 | 0.66 | 4 |  |
| *Cyathodes glauca* | GEF | -31.91 | 0.92 | 160.84 | 8.84 | 0.77 | 3.5 |  |
| *Eucalyptus delegatensis* | GEF | -31.96 | 29.63 | 186.86 | 3.88 | 0.53 | 87 | 0.030 |
| *Eucalyptus obliqua* | GEF | -31.04 | 40.98 | 179.39 | 3.91 | 0.62 | 90 | 0.022 |
| *Eucalyptus regnans* | GEF | -31.88 | 32.86 | 154.18 | 3.17 | 0.60 | 99.6 | 0.021 |
| *Gaultheria hispida* | GEF | -32.67 | 4.93 | 113.81 | 4.55 | 0.50 | 2 |  |
| *Melaleuca squarrosa* | GEF | -31.49 | 0.53 | 93.24 | 2.37 | 0.59 | 11 | 0.038 |
| *Monotoca glauca* | GEF | -32.88 | 1.73 | 132.50 | 4.31 | 0.63 | 6 |  |
| *Nematolepis squamea* | GEF | -31.26 | 5.71 | 122.03 | 5.93 | 0.77 | 10 | 0.061 |
| *Olearia argophylla* | GEF | -30.81 | 41.67 | 114.65 | 2.73 | 0.67 | 10 | 0.021 |
| *Olearia lirata* | GEF | -30.95 | 20.80 | 58.69 | 5.16 | 0.57 | 3 | 0.039 |
| *Oxylobium arborescens* | GEF | -31.41 | 1.92 | 143.79 | 5.17 | 0.70 | 3.2 | 0.058 |
| *Pimelea cinerea* | GEF | -34.05 | 2.12 | 92.13 | 2.97 | 0.52 | 2 |  |
| *Pimelea drupacea* | GEF | -34.61 | 4.23 | 56.65 | 4.10 | 0.43 | 3 |  |
| *Pittosporum bicolor* | GEF | -33.39 | 4.89 | 117.00 | 5.23 | 0.68 | 14 | 0.100 |
| *Pomaderris apetala* | GEF | -32.03 | 20.51 | 124.12 | 2.81 | 0.60 | 13 | 0.044 |
| *Prostanthera lasianthos* | GEF | -32.05 | 14.74 | 63.11 | 4.48 | 0.58 | 6 | 0.033 |
| *Zieria arborescens* | GEF | -30.53 | 7.59 | 64.48 | 4.01 | 0.77 | 6 | 0.070 |
| *Acacia genistifolia* | SAV | -30.29 | 0.23 | 286.95 | 7.16 | 0.69 | 1.2 |  |
| *Acacia myrtifolia* | SAV | -30.25 | 2.22 | 171.30 | 3.06 | 0.63 | 1 |  |
| *Acacia stricta* | SAV | -32.53 | 3.94 | 138.34 | 9.61 | 0.74 | 1.75 |  |
| *Astroloma humifusum* | SAV | -32.97 | 0.11 | 120.06 | 8.50 | 0.76 | 0.06 |  |
| *Banksia marginata* | SAV | -30.74 | 2.66 | 199.31 | 6.48 | 0.61 | 5 | 0.109 |
| *Bedfordia linearis* | SAV | -31.09 | 2.68 | 166.91 | 12.86 | 0.77 | 3 | 0.091 |
| *Bursaria spinosa* | SAV | -31.16 | 0.36 | 156.78 | 1.46 | 0.72 | 2.2 | 0.125 |
| *Callistemon pallidus* | SAV | -30.54 | 3.53 | 202.14 | 6.00 | 0.75 | 4 | 0.117 |
| *Correa reflexa* | SAV | -31.65 | 4.08 | 94.79 | 1.63 | 0.77 | 0.8 |  |
| *Epacris impressa* | SAV | -31.39 | 0.13 | 156.54 | 4.87 | 0.72 | 1.6 |  |
| *Eucalyptus pulchella* | SAV | -30.94 | 4.22 | 206.79 | 15.47 | 0.61 | 13 | 0.051 |
| *Eucalyptus viminalis* | SAV | -30.23 | 15.48 | 226.69 | 9.26 | 0.67 | 22 | 0.090 |
| *Hibbertia riparia* | SAV | -31.77 | 0.10 | 166.46 | 8.68 | 0.82 | 0.6 |  |
| *Leptecophylla juniperina* | SAV | -33.92 | 0.10 | 149.82 | 6.48 | 0.84 | 2 |  |
| *Leucopogon collinus* | SAV | -29.54 | 0.07 | 91.06 | 3.71 | 0.78 | 1.7 |  |
| *Lomatia tinctoria* | SAV | -30.97 | 0.31 | 188.72 | 8.35 | 0.78 | 0.55 |  |
| *Philotheca verrucosa* | SAV | -29.88 | 0.20 | 217.10 | 1.58 | 0.82 | 1.4 |  |
| *Pimelea nivea* | SAV | -30.27 | 0.59 | 144.09 | 1.06 | 0.53 | 1.5 |  |
| *Pultenaea juniperina* | SAV | -31.98 | 0.17 | 131.50 | 7.49 | 0.88 | 2.2 |  |
| *Veronica formosa* | SAV | -29.45 | 0.26 | 109.38 | 2.96 | 0.69 | 1.7 |  |

**Table S2.** Phylogenetic One-way ANOVA results for leaf and bole plant functional trait comparisons between rainforests, giant eucalypt forests and savannas of tropical and temperate regions. Bark thickness was excluded from this analysis as it consisted of only a subset of the species in the phylogenetic tree.

| **Functional Trait** | **Tropical Queensland** | **Temperate Tasmania** |
| --- | --- | --- |
| Delta 13 C (δ^13^C) | *F* = 6.97, *P* = 0.03* | *F* = 4.11, *P* = 0.02* |
| Leaf area | *F* = 16.31, *P* = 0.002** | *F* = 7.31, *P* = 0.002** |
| Leaf mass per area (LMA) | *F* = 20.56, *P* = 0.001** | *F* = 8.83, *P* = 0.0005*** |
| Leaf slenderness | *F* = 11.48, *P* = 0.004** | *F* = 0.0987, *P* = 0.0906 |
| Wood density | *F* = 6.63, *P* = 0.02* | *F* = 7.5959, *P* = 0.0012** |
| Maximum height | *F* = 4.31, *P* = 0.099 | F = 17.7665, P <0.0001*** |

**References**

1. Bell CD, Soltis DE, Soltis PS (2010) The age and diversification of the angiosperms re-revisited. Am J Bot 97: 1296–1303.
2. Quinn CJ, Crayn DM, Heslewood MM, Brown EA, Gadek PA (2003) A molecular estimate of the phylogeny of Styphelieae (Ericaceae). Aust Syst Bot 16: 581–594.
3. Biffin E, Lucas EJ, Craven LA, Ribeiro da Costa I, Harrington MG, et al. (2010) Evolution of exceptional species richness among lineages of fleshy-fruited Myrtaceae. Ann Bot 106: 79–93.
4. Sauquet H, Weston PH, Anderson CL, Barker NP, Cantrill DJ, et al. (2009) Contrasted patterns of hyperdiversification in Mediterranean hotspots. PNAS 106: 221–225.
5. Murphy DJ, Brown GK, Miller JT, Ladiges PY (2010) Molecular phylogeny of *Acacia* Mill. (Mimosoideae: Leguminosae): Evidence for major clades and informal classification. Taxon 59: 7–19.
6. Snow N, McFadden J, Evans TM, Salywon AM, Wojciechowski MF, et al. (2011) Morphological and Molecular Evidence of Polyphyly in *Rhodomyrtus* (Myrtaceae: Myrteae). Syst Bot 36: 390–404.
7. Steane DA, Nicolle D, Sansaloni CP, Petroli CD, Carling J, et al. (2011) Population genetic analysis and phylogeny reconstruction in *Eucalyptus* (Myrtaceae) using high-throughput, genome-wide genotyping. Molec Phylogen Evol 59: 206–224.
